# A potential method of identifying stroke and other intracranial lesions in a prehospital setting

**DOI:** 10.1186/s13049-020-00728-7

**Published:** 2020-05-13

**Authors:** Anssi Saviluoto, Heini Harve-Rytsälä, Mitja Lääperi, Hetti Kirves, Helena Jäntti, Jouni Nurmi

**Affiliations:** 1 Research and Development Unit, FinnHEMS, WTC Helsinki Airport, Lentäjäntie 3, FI-01530 Vantaa, Finland; 2 University of Eastern Finland, PO Box 1627, FI-70211 Kuopio, Finland; 3 Department of Emergency Medicine and Services, University of Helsinki and Helsinki University Hospital, PO Box 340, FI-00029 HUS, Finland; 4 Prehospital Emergency Care, Hyvinkää hospital area, Hospital District of Helsinki and Uusimaa, PO Box 585, FI-05850 Hyvinkää, Finland; 5 Center for Prehospital Emergency Care, Kuopio University Hospital, PO Box 100, FI-70029 Kuopio, Finland

**Keywords:** Blood pressure, Heart rate, Age, Stroke, Emergency medical services, Intracranial pressure, Intubation, intratracheal

## Abstract

**Background:**

Identifying stroke and other intracranial lesions in patients with a decreased level of consciousness may be challenging in prehospital settings. Our objective was to investigate whether the combination of systolic blood pressure, heart rate and age could be used to identify intracranial lesions.

**Methods:**

We conducted a retrospective case-control study including patients with a decreased level of consciousness who had their airway secured during prehospital care. Patients with intracranial lesions were identified based on the final diagnoses at the end of hospitalization. We investigated the ability of systolic blood pressure, heart rate and age to identify intracranial lesions and derived a decision instrument.

**Results:**

Of 425 patients, 127 had an intracranial lesion. Patients with a lesion were characterized by higher systolic blood pressure, lower heart rate and higher age (*P* < 0.0001 for all). A systolic blood pressure ≥ 140 mmHg had an odds ratio (OR) of 3.5 (95% confidence interval [CI] 1.7 to 7.0), and > 170 mmHg had an OR of 8.2 (95% CI 4.5–15.32) for an intracranial lesion (reference: < 140 mmHg). A heart rate < 100 beats/min had an OR of 3.4 (95% CI 2.0 to 6.0, reference: ≥100). Age 50–70 had an OR of 4.1 (95% CI 2.0 to 9.0), and > 70 years had an OR of 10.2 (95% CI 4.8 to 23.2), reference: < 50. Logarithms of ORs were rounded to the nearest integer to create a score with 0–2 points for age and blood pressure and 0–1 for heart rate, with an increasing risk for an intracranial lesion with higher scores. The area under the receiver operating characteristics curve for the instrument was 0.810 (95% CI 0.850–0.890).

**Conclusions:**

An instrument combining systolic blood pressure, heart rate and age may help identify stroke and other intracranial lesions in patients with a decreased level of consciousness in prehospital settings.

**Trial registration:**

Not applicable.

## Background

Patients with an altered level of consciousness are often encountered by emergency medical services (EMS) in the prehospital setting and by emergency department (ED) personnel in-hospital [1, 2]. Common causes of altered levels of consciousness include epilepsy, hypoglycemia and intoxication by alcohol or other substances [2]. Obvious causes for a decreased level of consciousness (e.g., hypoglycemia) can be ruled out in the prehospital setting, but often the cause remains unknown [2]. An intracranial lesion, e.g., a stroke, is a frequent cause of a decreased level of consciousness [3, 4]. and should be recognized as early as possible to avoid a delay in transport to an optimal tertiary-care unit with appropriate recanalization and neurosurgical capabilities [5, 6]. Several scoring systems have been developed to recognize ischemic stroke and especially large vessel occlusions [7, 8]. However, these scores rely on clinical findings that may be impossible to examine on a patient with a markedly decreased level of consciousness. Identifying the patients with increased intracranial pressure (ICP) would also enable the utilization of neuroprotective methods during anesthesia and intubation frequently performed to secure the airway of unconscious patients [9].

Many patients with an intracranial lesion are hypertensive [3, 10]. because of the Cushing reflex [11, 12]. or central nervous system ischemic response [13, 14]. Previous studies have shown that blood pressure and pulse can be useful indicators when discerning whether the patient is suffering from an intracranial lesion causing an altered level of consciousness [3, 4]. Age has also been shown to correlate with an increased risk for intracranial lesions [4].

We hypothesized that the first blood pressure and pulse measured in the prehospital setting, combined with the age of the patient, could be used to predict if a patient with an altered level of consciousness has an intracranial lesion.

## Methods

### Study design

We conducted a retrospective case-control study comparing the initial prehospital systolic blood pressure, heart rate and age of patients with and without an intracranial lesion. The study was reported according to the Strengthening the Reporting of Observational Studies in Epidemiology (STROBE) statement. The study was retrospective and register-based. The data was de-identified before analysis. Patients were not contacted for study purposes, nor did the study affect their treatment. Thus, approval from an ethics committee was not required by Finnish legislation. The authorities of the Helsinki University Hospital approved the study protocol and provided permission to access the patient data.

### Setting and population

The study was based on the FinnHEMS database, where all the missions of every helicopter emergency medical services (HEMS) unit in Finland are entered. We included only patients of the busiest HEMS unit of the country, as carefully validated data of intubated patients were available [15]. The data were entered after the mission by the physician in charge of each patient’s care. The dataset also included the first vital signs of the patient, measured by the first EMS unit before arrival of the HEMS unit.

We analyzed the data of patients with Glasgow Coma Score < 15, having their airway managed by the crew of a single HEMS unit (FinnHEMS 10) and alive upon arrival at the hospital during 2014 and between March 2015 and Dec 2016. Data on patients encountered during Jan 2015 and Feb 2015 were not used because of the changes in the operational models of the HEMS unit during that time, which would have potentially biased the quality of the registry data. The HEMS unit is dispatched as an addition to ground units by emergency dispatchers on predetermined criteria. It is alerted for all missions where an unconscious patient without a pain response is reported by the caller. The only exemptions are suspected hypoglycemia or convulsions, where only a paramedic staffed unit is dispatched. At the time of the study, the HEMS unit was the only EMS unit providing prehospital anesthesia and intubation in its operation area. Thus, the unit covers virtually every unconscious patient in need of airway management.

All adult (age > 16) patients with their airway managed by the HEMS crew and patients with a recorded GCS < 15, were included. The indications and protocol for prehospital anesthesia in the unit are described in a previous study [15]. Patients under 16 years of age were excluded because the normal blood pressure of a child varies according to age [16]. Patients with out-of-hospital cardiac arrest as the primary reason for the mission were excluded because of post cardiac arrest syndrome, including myocardial stunning, hemodynamics and the level of consciousness. Those with an obvious traumatic etiology assessed by the HEMS physician on scene were excluded because the reason for the decreased level of consciousness was obvious.

### Measurements and variables

For the study, we used the first systolic blood pressure and heart rate acquired by the first EMS unit on scene. Blood pressure and heart rate were measured using a monitor-defibrillator with an automatic noninvasive blood pressure monitor (Lifepak 12 or 15 by Physio-Control Inc. Redmond,WA, USA or Zoll M or X series by Zoll Medical Corporation, Chelmsford, MA, USA). The measurements were automatically transferred via WiFi or Bluetooth to the electronic patient record system, removing the potential of errors in data collection. Patients with either heart rate or blood pressure recorded initially were analyzed for the recorded vital sign. All patients in the area were transported to the public hospitals with the electronic patient record system. The primary diagnoses according to the International Statistical Classification of Diseases (ICD-10) at the end of hospitalization period were searched afterwards from the hospital’s electronic patient record system (Uranus® CGI Suomi Oy, Finland).

Included patients were categorized into two groups according to their final diagnoses at the end of hospitalization: 1) those having an intracranial lesion and 2) those without. In addition to stroke, other diagnoses that may lead to elevation of ICP were included in the former group [12]. A full list of diagnoses categorized as intracranial lesions can be seen in Additional file 1. Seizures due to epilepsy, status epilepticus, alcohol withdrawal or other unclassified convulsions were not categorized as intracranial lesions (ICD-10 categories G40-G41, R56.8, F10.31).

### Statistical analysis

The two groups were compared by age, heart rate and systolic blood pressure. We divided each variable into categories to derive a scoring system for clinical use. Finally, the diagnostic accuracy of the scoring system to detect an intracranial lesion was tested. Based on our data, we chose convenient cut-off values of 140 and 170 mmHg for systolic blood pressure. The lower cut-off for blood pressure was the cut-off for stage 2 hypertension according to the AHA guidelines [17]. The upper cut-off of 170 mmHg was chosen because it has been shown to correlate with an increased likelihood for an intracranial lesion [3]. and correlate with worse outcomes in ischemic stroke [18]. For age, we chose cut-offs of 50 and 70, and for heart rate, we chose a common definition of tachycardia of 100 beats/min [19]. as a cutoff.

Independent two-sample t-tests for equal variances were used to determine the significance of differences in blood pressure, heart rate and respiratory rate between the groups. For age and peripheral oxygen saturation, independent two-sample t-tests for unequal variances were used. For age, we used the χ2 test.

We investigated the predictive ability of systolic blood pressure, age and heart rate to predict whether a patient would have a lesion. We fitted logistic regression models for the variables separately and with all three in the same model. The multivariable model was then used to create a score for the lesions.

The discrimination abilities of the models were investigated using receiver operating characteristics curves (ROC) and areas under the ROC (AUROC) [20]. We also used the Loess method to visualize the performance of the score. The calibrations of the models were investigated both visually and using Hosmer-Lemeshow [21]. tests. All analyses were carried out using R version 3.5.1 [22]. and packages ggplot2 [23]., pROC [20]. and ResourceSelection [21]. As a separate sensitivity analysis, we experimentally excluded all patients with a primary diagnosis of epilepsy or other seizures (as defined above).

## Results

**Fig. 1:**
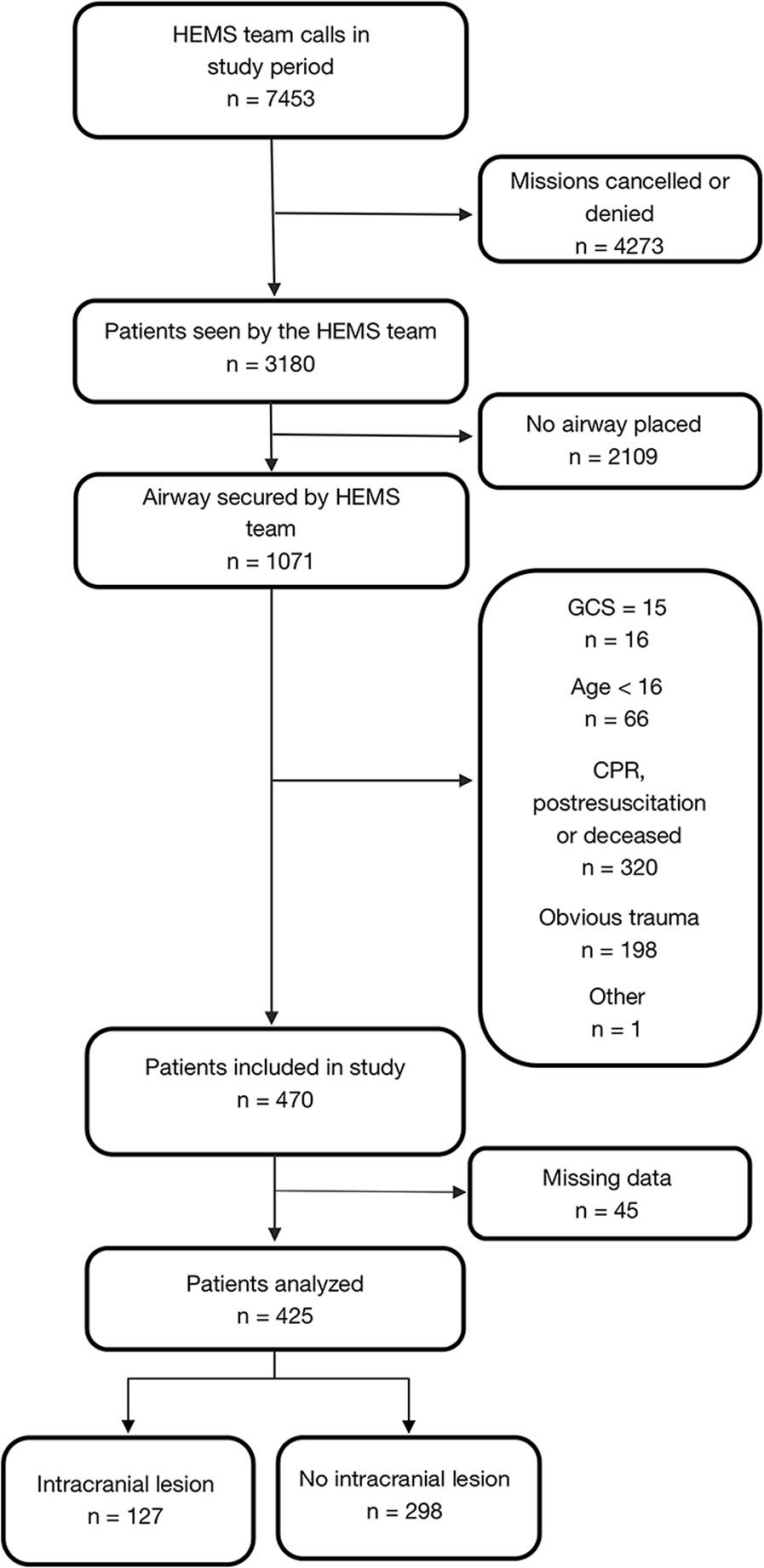
Patient selection flowchart

During the study period, 1071 patients had their airway secured by the HEMS crew. After exclusions, 425 patients were analyzed in the study (Fig. 1). By the diagnoses at the end of hospitalization, 127 (30%) subjects were categorized as having an intracranial lesion and 298 (70%) as not having a lesion. A single patient had a primary diagnosis of concussion (S06.0) without any other diagnoses reported. We found this case difficult to categorize in either group, so it was excluded from the analysis.

Of patients with an intracranial lesion, 98 (77%) had a stroke, including 41 with intracerebral hemorrhages (I61), 31 with subarachnoidal hemorrhages (I60) and 21 with cerebral infarctions (I63). A total of 21 (17%) were found to have an intracranial injury (S06.1-S06.9), with 18 traumatic subdural hemorrhages (S06.5). Eight (6%) were found to have other intracranial lesions, of which 5 were inflammatory diseases of the central nervous system (G00–09).

Of the patients without an intracranial lesion, 92 (31%) had a diagnosis of poisoning by drugs, medicaments and biological substances (T36-T50), 83 (28%) had a primary diagnosis of epilepsy, status epilepticus or unspecified convulsions (G40-G41, R56.9), and 123 (41%) had other diagnoses.

Patients with an intracranial lesion were characterized by higher systolic blood pressure, lower heart rate and higher age (Table 1).

**Table 1:** Patient characteristics of the patients with and without intracranial lesions. Values are presented as median (interquartile range [range]) or number (proportion)

	Intracranial lesion	No lesion	*P*-value
	*N* = 127	*N* = 298	
Age	69 (60–77 [24–97])	51 (33–66 [16–93])	< 0.0001
Sex;male	61 (48%)	168 (56%)	0.114
Initial systolic blood pressure	176 (145–198 [67–256])	126 (105–146 [55–270])	< 0.0001
Initial heart rate	81 (66–106 [41–154])	98(77–117[20–207])	< 0.0001
Initial Glasgow Coma Scale	5(3–6[3–15])	4(3–7[3–15])	0.754
Initial peripheral capillary oxygen saturation	94(88–97[52–100])	93(86–97[40–100]]	0.12
Initial respiratory rate	18(13–26[0–45])	18(12–25[0–96])	0.865

Distinguishing between patients with and without an intracranial lesion, initial systolic blood pressure had an AUROC of 0.808 (95% CI 0.762 to 0.853). The initial heart rate and age had AUROCs of 0.616 (95% CI 0.558 to 0.675) and 0.769 (95% CI 0.724 to 0.814), respectively.

Heart rate was divided into two categories, while age and systolic blood pressure were grouped into three categories (Table 2). The regression model results for continuous and categorized variables can be seen in Additional file 2. All of the models calibrated well (*p*-value n.s.). The AUROC for the continuous multivariate model was 0.858 (95% CI 0.819 to 0.896) and was 0.852 (95% CI 0.813 to 0.892) for the categorized model.

**Table 2:** Odds ratios of blood pressure, heart rate and age categories for intracranial lesions and HeSA-scoring system conducted

Variable	HeSA-Score Points*	OR for an intracranial lesion	95% CI	*P*-value
**Systolic blood pressure**				
< 140 mmHg^†^	0	1		
140–170 mmHg	1	3.5	1.7–7.0	< 0.001
> 170 mmHg	2	8.2	4.5–15.3	< 0.001
**Heart rate**				
≥ 100 / min^†^	0	1		
< 100 / min	1	3.4	2.0–6.0	< 0.001
**Age**				
< 50 years^†^	0	1		
50–70 years	1	4.1	2.0–9.0	< 0.001
> 70 years	2	10.2	4.8–23.2	< 0.001

^(^*^)^ Points are log (OR) estimates (†) Reference

The score was created by counting logarithms of odds ratios from the multivariable model (Table 2). These were rounded to the closest integer and used as points in the decision instrument (Table 2).

**Fig. 2:**
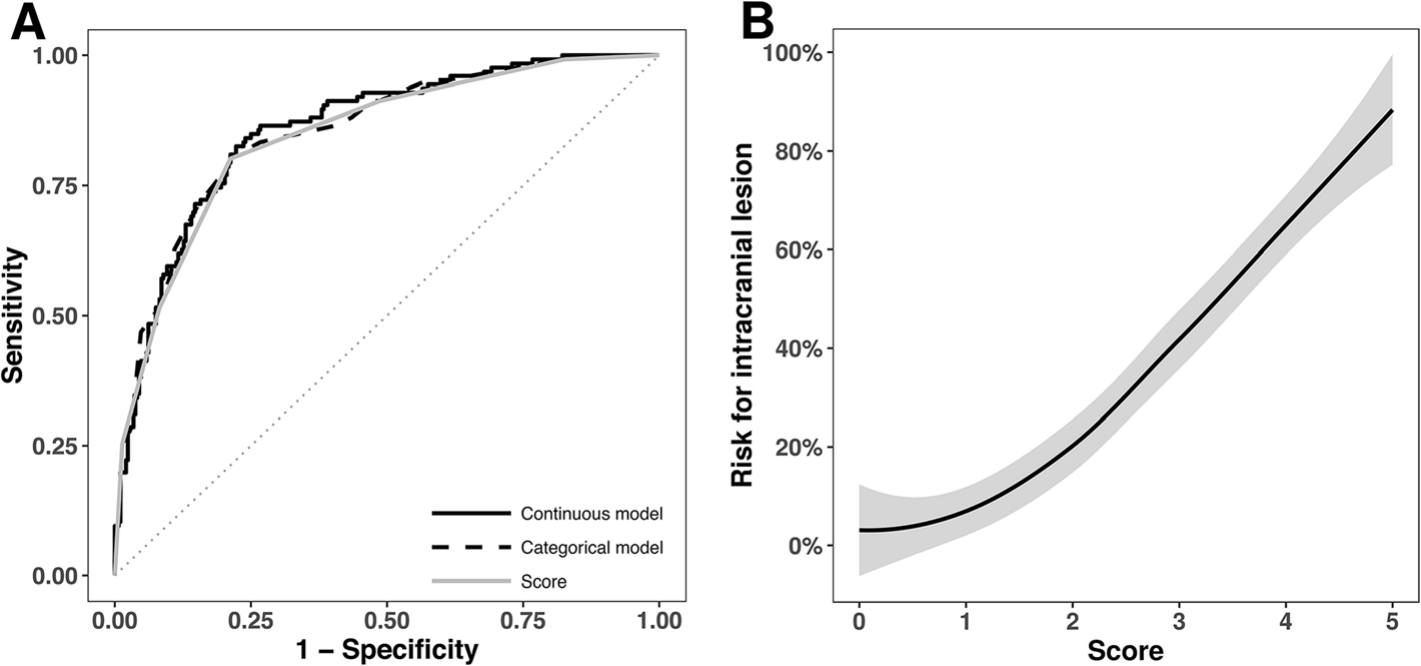
HeSA-score performance in detecting intracranial lesions presented as (**a**) receiver operating curve for multivariate model with comparison with noncategorized model and (**b**) Loess curve with 95% confidence interval marked in gray

The resulting score discriminated well between having and not having an intracranial lesion: the AUROC was 0.810 (95% CI 0.850 to 0.890), Fig. 2. The distribution of the patients within the scoring system along with properties of different cut-offs are presented in Table 3.

**Table 3:** Characteristics of scoring system and patient count at different cut-offs

	1 point	2 points	3points	4 points	5 points
Number of patients	109	94	74	53	36
Sensitivity (%)	99	91	80	52	25
Specificity (%)	17	51	79	92	99
Positive predictive value (%)	34	45	62	73	89
Negative predictive value (%)	98	93	90	81	75

Excluding all patients with a primary diagnosis of epilepsy or other seizures in a separate sensitivity analysis further improved the discrimination capability of the score, resulting in an AUROC of 0.844 (95% CI 0.882–0.920).

## Discussion

Our study indicated that a decision instrument combining the initial systolic blood pressure, heart rate and age can provide a useful aid in assessing whether a decrease in the level of consciousness is caused by an intracranial lesion or some other mechanism. To our knowledge, this is the first study investigating this approach in a prehospital patient population. Our findings are consistent with previous studies performed in Japanese EDs [3, 4]. Ikeda M et al. [3]. found systolic blood pressure to have an AUROC of 0.9 within a slightly elderly population, including more patients with stroke than the current study. Furthermore, in line with our findings, Yamashiro et al. [4]. reported systolic blood pressure and age to have the best informative usefulness, with heart rate being marginally predictive. It is also noteworthy that our study population had a high incidence of intoxications compared to previous studies.

We did not categorize seizures as intracranial lesions, as the standard of the care does not include measures to control intracranial hypertension, in contrast to the care of patients with intracranial lesions such as a stroke [24]. Furthermore, these patients are typically managed in hospitals other than neurocritical centers [25]. Therefore, their prehospital care resembles more than that of the patients with no intracranial lesion. Because this categorization was in contrast to the previous studies by Yamashiro et al. [4]. and Ikeda et al. [3]., we experimentally performed a separate analysis excluding all 89 patients with a diagnosis of seizures from the analysis, which resulted in a modest improvement of the diagnostic accuracy of the score.

There is a strong rationale underlying the use of blood pressure, heart rate and age to detect intracranial lesions in unconscious patients. As previously stated, an increase in blood pressure and a decrease in heart rate are commonly observed during stroke [10]. as a consequence of Cushing reflex [11, 12]. and ischemic response [13, 14]. In contrast, common extracranial causes such as sepsis and intoxication by sedatives tend to cause hypotension [26–28]. The incidence of stroke increases substantially with increasing age [4].

Identifying patients with stroke or other intracranial lesions early is paramount because the focus of prehospital care of these patients is to minimize a secondary insult to the brain tissue and maximize salvageable brain by optimizing brain perfusion and oxygenation while expediently transporting the patients to a stroke unit or neurosurgical center [29]. Several scoring systems have been devised to recognize patients with an ischemic stroke [8]. Also, many decision instruments have been created to identify large vessel occlusion in the prehospital setting [7]. However, these instruments are not designed to recognize other intracranial lesions. Moreover, these instruments rely on neurological findings such as facial palsy, eye movement, speech impairment and asymmetries in the motor function of upper or lower extremities [7, 8]., all being impossible to detect on a patient with a markedly decreased level of consciousness. When prehospital anesthesia is necessary, neuroprotective measures should be applied to mitigate the possible deleterious effects of laryngoscopy and intubation [9]. There is substantial evidence that dedicated stroke centers decrease death, dependency and institutionalization [30]. Other causes of a decreased level of consciousness, such as intoxication, infection, epilepsy and substance withdrawal, focus on specific treatments and do not commonly include intensive ICP management. The vast majority of these patients do not need direct transport to a tertiary care center but can be treated in other hospitals instead [25].

We believe that in the case of these high priority calls, prehospital personnel could use this instrument as guidance when deciding on the receiving hospital and whether to engage in procedures to control ICP. We named the HeSA-score according to the measured attributes: heart rate, systolic blood pressure and age. Using the scoring system is simple, and a score ≥ 2 provides a good sensitivity of over 0.9 for an intracranial lesion, while a score of 3 provides a useful combination of sensitivity (0.802) and specificity (0.788). At higher scores, our tool becomes highly specific. The score could be integrated into the electronic patient record system, providing an automatic warning when detecting a patient at an elevated likelihood for having an intracranial lesion. In some regions, the utilization of mobile stroke units has brought CT capabilities to the prehospital setting [31], but because of the costs and range of these units, they are not recently widely available in suburban and rural areas. Our decision instrument might also prove helpful when prioritizing to which call the mobile stroke unit is assigned.

### Limitations

The main limitation of the study is that the categorization was performed using the diagnoses acquired from patient records, and the validity and uniformity of the diagnosis processes were not controlled by any study protocol. Not all the diagnoses were confirmed by advanced imaging of the brain; thus, some patients categorized as not having an intracranial lesion might have had a lesion that remained undiagnosed. However, all the hospitals to which the studied patients were transported had resources to perform instant computer tomography imaging, and most also had magnetic resonance imaging scans available at least during the daytime. Thus, the reason not to confirm the diagnosis by advanced imaging has not been the non-availability of the imaging but a clinical decision instead. All hospitals receiving and treating intubated patients at the time of the study were also part of the public health care system, funded by municipalities and the state. Thus, the social status or health insurance of the patient did not have any effect on the investigations or treatments. The diagnoses used in the study were set at the end of hospitalization. Thus, we assume that cases where an underlying lesion remained unrecognized were rare in the study population.

The study population consisted of patients requiring airway management; therefore, further study is needed to investigate whether the score could be applied to more well-appearing patients. The score was developed based on the patients treated by a single HEMS-unit, the busiest base in the country, creating a possible source of bias. Therefore, validation in other patient populations is necessary. We are planning further studies including data from all six Finnish HEMS units. However, the strength of the study is that it included data from virtually all unconscious patients in need of intubation in the study area. The HEMS unit does not have strict criteria on the decision-making process if the patient needs intubation, and the final conclusion is made by the anesthesiologist on the scene. Thus, selection bias is possible but unlikely.

## Conclusions

Patients with altered level of consciousness caused by stroke and other intracranial lesion can be identified by the combination of systolic blood pressure, heart rate and age. This approach complements the current scoring systems that require co-operation of the patient to neurological examination.

## Supplementary information

**Additional file 1.** Diagnoses categorized as intracranial lesions

**Additional file 2.** Regression analysis results

## Data Availability

The anonymized data that support the findings of this study are available from the corresponding author upon reasonable request.

## Abbreviations

EMS: Emergency medical services
ED: Emergency department
ICP: Intracranial pressure
STROBE: Strengthening the Reporting of Observational Studies in Epidemiology
HEMS: Helicopter emergency medical services
GCS: Glasgow Coma Scale
ICD-10: International Statistical Classification of Diseases
AHA: American Heart Association
ROC: Receiver operating characteristics curve
AUROC: Area under receiver operating characteristics curve
CT: Computed tomography
MSU: Mobile stroke unit

## References

[1] Sanello A, Gausche-Hill M, Mulkerin W, Sporer KA, Brown JF, Koenig KL (2018). Altered mental status: current evidence-based recommendations for Prehospital care. West J Emerg Medicine.

[2] Björkman J, Hallikainen J, Olkkola KT, Silfvast T (2016). Epidemiology and aetiology of impaired level of consciousness in prehospital nontrauma patients in an urban setting. Eur J Emerg Med.

[3] Ikeda M, Matsunaga T, Irabu N, Yoshida S (2002). Using vital signs to diagnose impaired consciousness: cross sectional observational study. Bmj..

[4] Yamashiro S, Oda Y, Kanegae S, Shirahama M, Yoshihara K, Fukui T (1994). Informative usefulness of age, sex and vital signs in the differential diagnosis of disturbed consciousness among 175 emergency outpatients. Fukuoka igaku zasshi.

[5] Lachkhem Y, Rican S, Minvielle É (2018). Understanding delays in acute stroke care: a systematic review of reviews. Eur J Pub Health.

[6] Langhorne P, Fearon P, Ronning OM, Kaste M, Palomaki H, Vemmos K (2013). Stroke unit care benefits patients with Intracerebral hemorrhage. Stroke..

[7] Vidale S, Agostoni E (2018). Prehospital stroke scales and large vessel occlusion: a systematic review. Acta Neurol Scand.

[8] Rudd M, Buck D, Ford GA, Price CI (2016). A systematic review of stroke recognition instruments in hospital and prehospital settings. Emerg Med J.

[9] von Elm E, Schoettker P, Henzi I, Osterwalder J, Walder B (2009). Pre-hospital tracheal intubation in patients with traumatic brain injury: systematic review of current evidence. Brit J Anaesth.

[10] Wallace J, Levy L (1981). Blood pressure after stroke. JAMA..

[11] Cushing H (1901). Concerning a definite regulatory mechanism of the Vaso-motor Centre which controls blood pressure during cerebral compression. Johns Hopkins Hosp Bull.

[12] Freeman DW (2015). Management of Intracranial Pressure. Continuum Lifelong Learn Neurology.

[13] Reis DJ, Golanov EV, Galea E, Feinstein DL (1997). Central neurogenic Neuroprotection: central neural systems that protect the brain from hypoxia and ischemia. Ann N Y Acad Sci.

[14] Hall J. Cushing Reaction to Increased Pressure Around the Brain. In:Hall JE, Guyton CA. Guyton and Hall textbook of medical physiology. 13th ed. Philadelphia, USA: Elsevier, 2016. p. 223.

[15] Ångerman S, Kirves H, Nurmi J (2018). A before-and-after observational study of a protocol for use of the C-MAC videolaryngoscope with a Frova introducer in pre-hospital rapid sequence intubation. Anaesthesia..

[16] Flynn JT, Kaelber DC, Baker-Smith CM, Blowey D, Carroll AE, Daniels SR (2017). Clinical practice guideline for screening and Management of High Blood Pressure in children and adolescents. Pediatrics..

[17] Whelton M, Carey FM, Aronow FS, Jr ME, Collins WA. Himmelfarb, et al. 2017 ACC/AHA/AAPA/ABC/ACPM/AGS/APhA/ASH/ASPC/NMA/PCNA guideline for the prevention, detection, evaluation, and Management of High Blood Pressure in adults: executive summary. J Am Coll Cardiol. 2017;71:1–114. 10.1016/j.jacc.2017.11.006 29146535

[18] Stead L, Enduri S, Bellolio FM, Jain AR, Vaidyanathan L, Gilmore RM (2012). The impact of blood pressure hemodynamics in acute ischemic stroke: a prospective cohort study. Int J Emerg Medicine.

[19] Hall J. Abnormal sinus rhythms. In: Hall JE, Guyton CA. Guyton and Hall textbook of medical physiology. 13th ed. Philadelphia: Elsevier; 2016. p. 155.

[20] Robin X, Turck N, Hainard A, Tiberti N, Lisacek F, Sanchez J-C (2011). pROC: an open-source package for R and S+ to analyze and compare ROC curves. Bmc Bioinformatics.

[21] Lele S, Keim J, Solymos P (2017). Resource selection (probability) functions for use-availability data.

[22] http www org TR, 2018. R: a language and environment for statistical computing. R Foundation for Statistical Computing V, Austria 2016.

[23] Wickham H. ggplot2: elegant graphics for data analysis. 2016.

[24] Billington M, Kandalaft OR, Aisiku IP (2016). Adult status Epilepticus: a review of the Prehospital and emergency department management. J Clin Medicine.

[25] Howard RS, Kullmann DM, Hirsch NP. Admission to neurological intensive care: who, when, and why? J Neurology Neurosurg Psychiatry. 2003;74:iii2-iii9. 10.1136/jnnp.74.suppl_3.iii2 PMC1765634 12933908

[26] Angus DC, van der Poll T (2013). Severe Sepsis and septic shock. New Engl J Medicine.

[27] Vonghia L, Leggio L, Ferrulli A, Bertini M, Gasbarrini G, Addolorato G (2008). Acute alcohol intoxication. Eur J Intern Med.

[28] Ashton H (1995). Toxicity and adverse consequences of benzodiazepine use. Psychiat Ann.

[29] Haddad SH, Arabi YM (2012). Critical care management of severe traumatic brain injury in adults. Scand J Trauma Resusc Emerg Medicine.

[30] Collaboration S. Organised inpatient (stroke unit) care for stroke. Cochrane Db Syst Rev. 2013;23 Suppl 2:CD000197.

[31] John S, Stock S, Cerejo R, Uchino K, Winners S, Russman A (2016). Brain imaging using Mobile CT: current status and future prospects. J Neuroimaging.

