# Supplementary material for: A potential method of identifying stroke and other intracranial lesions in a prehospital setting
Source: Scand J Trauma Resusc Emerg Med. 2020 May 13;28:39. doi: 10.1186/s13049-020-00728-7 (PMC7222442; doi:10.1186/s13049-020-00728-7)
Supplement: Supplementary file 1 — Additional file 1. Diagnoses categorized as intracranial lesions [file 13049_2020_728_MOESM1_ESM.docx]

Additional file 1

Diagnoses categorized as intracranial lesions

| ICD-10 categorization | Diagnosis | n |
| --- | --- | --- |
| I61 | Intracerebral haemorrhage | 41 |
| I60 | Subarachnoid haemorrhage | 31 |
| I63 | Cerebral infarction | 21 |
| S06.5 | Traumatic subdural haemorrhage | 18 |
| I64 | Stroke, not specified as haemorrhage or infarction | 3 |
| S06.3 | Focal brain injury | 2 |
| I62 | Other nontraumatic intracranial haemorrhage | 2 |
| G00 | Bacterial meningitis, not elsewhere classified | 2 |
| G91 | Hydrocephalus | 2 |
| S06.6 | Traumatic subarachnoid haemorrhage | 1 |
| G01 | Meningitis in bacterial diseases classified elsewhere | 1 |
| G04 | Encephalitis, myelitis and encephalomyelitis | 1 |
| G05 | Encephalitis, myelitis and encephalomyelitis in diseases classified elsewhere | 1 |
| C79.3 | Secondary malignant neoplasm of brain and cerebral meninges | 1 |
| S06.1 | Traumatic cerebral oedema | 0 |
| S06.2 | Diffuse brain injury | 0 |
| S06.4 | Epidural haemorrhage | 0 |
| S06.7 | Intracranial injury with prolonged coma | 0 |
| S06.8 | Other intracranial injuries | 0 |
| S06.9 | Intracranial injury, unspecified | 0 |
| C70 | Malignant neoplasm of meninges | 0 |
| C71 | Malignant neoplasm of brain | 0 |
| D32.0 | Benign neoplasm of meninges | 0 |
| D33.0 | Benign neoplasm: Brain, supratentorial | 0 |
| D33.1 | Benign neoplasm: Brain, infratentorial | 0 |
| D33.2 | Benign neoplasm: Brain, unspecified | 0 |
| D43.0 | Neoplasm of uncertain or unknown behaviour: Brain, supratentorial | 0 |
| D43.1 | Neoplasm of uncertain or unknown behaviour: Brain, infratentorial | 0 |
| D43.2 | Neoplasm of uncertain or unknown behaviour: Brain, unspecified | 0 |
| G02 | Meningitis in other infectious and parasitic diseases classified elsewhere | 0 |
| G03 | Meningitis due to other and unspecified causes | 0 |
| G06 | Intracranial and intraspinal abscess and granuloma | 0 |
| G07 | Intracranial and intraspinal abscess and granuloma in diseases classified elsewhere | 0 |
