# Supplementary material for: A potential method of identifying stroke and other intracranial lesions in a prehospital setting
Source: Scand J Trauma Resusc Emerg Med. 2020 May 13;28:39. doi: 10.1186/s13049-020-00728-7 (PMC7222442; doi:10.1186/s13049-020-00728-7)
Supplement: Supplementary file 2 — Additional file 2. Regression analysis results [file 13049_2020_728_MOESM2_ESM.docx]

Additional file 2

Regression analysis results

| **Univariate model** | OR for intracranial lesion | 95% CI | P-value |
| --- | --- | --- | --- |
| Systolic blood pressure per 1 mmHg increase | 1.031 | 1.025-1.038 | <0.0001 |
| Heart rate per 1 beat per minute increase | 0.987 | 0.979-0.994 | <0.0001 |
| Age per 1 year increase | 1.065 | 1.049-1.083 | <0.0001 |
| **Multivariate model** |  |  |  |
| Continuous model (95% CI) |  |  |  |
| Systolic blood pressure per 1 mmHg increase | 1.025 | 1.018-1.032 | <0.0001 |
| Heart rate per 1 beat per minute increase | 0.984 | 0.974-0.993 | <0.001 |
| Age per 1 year increase | 1.048 | 1.030-1.067 | <0.0001 |
|  |  |  |  |
